# A Preliminary Evaluation of Limb Salvage Surgery for Osteosarcoma around Knee Joint

**DOI:** 10.1371/journal.pone.0033492

**Published:** 2012-03-23

**Authors:** Xing Wu, Zheng-dong Cai, Zheng-rong Chen, Zhen-jun Yao, Guang-jian Zhang

**Affiliations:** 1 Department of Orthopaedics, Shanghai Tenth People's Hospital, Tongji University School of Medicine, Shanghai, China; 2 Department of Orthopaedics, Shanghai Zhongshan Hospital, Fudan University School of Medicine, Shanghai, China; Ospedale Pediatrico Bambino Gesù, Italy

## Abstract

**Objective:**

To evaluate the effectiveness and drawbacks of diversified procedures of limb salvage surgery (LSS), providing a reference of rational surgical criterion of LSS.

**Methods:**

Fifty eight patients with stage IIB extremity osteosarcoma around knee joint area between 1992 and 2002 were studied retrospectively. Among them, 43 patients were treated by LSS followed by reconstruction. Reconstruction approaches included re-implantation of irradiation-devitalized tumor bone (n = 12), autoclaving-devitalized tumor bone (n = 8), prosthetic replacement (n = 11), allograft transplantation (n = 8) and vascularized fibula autograft implantation (n = 4). Amputations were performed in 15 patients. Patients were followed up for 6–16 years.

**Results:**

There were no significant difference between LSS and amputation groups regarding disease free survival and local recurrence rates. The actuarial 5-year continuous disease free survival and local recurrence rate were 30.0% and 25.0% in patients of devitalized LSS group, whereas those were 56.5% and 8.7% in patients of non-devitalized reconstruction group. The complication rate was significantly higher in LSS group compared to amputation group (P = 0.003).

**Conclusion:**

LSS with non-devitalized procedures is the optimal treatment for osteosarcoma around knee joint area. Prosthesis implantation is the preferred option for bone reconstruction following LSS. Prevention and treatment of post-operative complications should be paid more attention to get good long-term outcomes of surgery.

## Introduction

The survival rate of osteosarcoma has been significantly improved with the advent of many effective chemotherapeutic drugs since the late 1970s [1], [2]; meanwhile, limb salvage surgery (LSS) has gradually become the mainstay treatment for osteosarcoma [3], [4] because of its functional and physiological benefits over traditional amputative procedures. Besides, LSS has also greatly improved the life quality and enhanced the courage of patients. However, the question of ‘what modus of LSS is optimal for patient in terms of effectiveness and economy?’ still remains perplexing for most of surgeons as miscellaneous procedures of LSS for osteosarcoma have been widely applied and reported all over the world in recent years. Few studies have been conducted so far to explore and evaluate the criteria for the options of performing limb salvage based on the long-term outcomes after surgery. These outcomes include but not limited to the disease-free survival(DFS), local recurrence rates, and postoperative complications of patients.

In this study, we retrospectively analyzed the surgical outcomes of 58 patients with osteosarcoma around knee joint who were treated in our hospital from February 1992 to December 2002, and attempted to evaluate and compare the effectiveness and drawbacks of the diversified procedures of LSS in terms of DFS, local recurrence rate and postoperative complications of patients with the aim to provide a reference of rational surgical criterion of LSS for patients.

## Materials and Methods

### Clinical characteristic

In total, 58 patients (30 males, 28 females) aged 12–55 years (median age 20.26 years) with pre-operatively or pathologically confirmed malignant primary osteosarcoma at the particular knee joint area were enrolled in this study. The sites of osteosarcoma included the distal femur (n = 30), proximal tibia (n = 24), and proximal fibula (n = 4).

The histological subtypes of the cases in our study were classified as osteoblastic type (n = 42), fibroblastic type (n = 10), chondroblastic type (n = 4) and other type (n = 2). All 58 patients were diagnosed as Enneking stage IIB disease and without local and distal metastasis at admission.

### Treatment

Surgical procedures: All 58 patients underwent surgical operations, and of the 43 patients who received LSS, wide resection of tumors was performed based on the Enneking staging system. A ‘wide resection’ refers to removal of the tumor and surrounding cuff (3–5 cm circumference of tumor-free resection margins) of normal tissue (which was verified by pathological section) [5]. Two major methods were used for reconstruction: devitalized approach including re-implantation of irradiation-devitalized tumor bone (extracorporeally irradiated for 30 min using 30–50 Gy high-energy x-ray produced by a linear accelerator; irradiation subgroup, n = 12) and re-implantation of autoclaved-devitalized tumor bone (extracorporeally boiled for 30 min; autoclaving subgroup, n = 8), and non-devitalized approach including prosthetic replacement (hinged knee prosthesis; prosthetic subgroup, n = 11), allograft transplantation (allograft subgroup, n = 8), and vascularized fibula autograft implantation (autograft subgroup, n = 4). Besides, amputation were operated in another 15 patients (amputation group, n = 15).

Chemotherapy: Patients in both LSS and amputation group received the same protocol of neoadjuvant chemotherapy, which referred to the Bacci (IOS/OS4) regimen [6], along with the surgery. The main drugs used included adriamycin, cisplatin, high dose methotrexate, and cyclophosphamide. Four cycles of protocols of adjuvant chemotherapy were administered preoperatively and two weeks after operation.

### Postoperative outcome evaluation and statistical analysis

All 58 patients were divided into 3 groups based on the treatment they received: G1 (Group 1), amputation group; G2 (Group 2), devitalized LSS group: LSS with reconstruction approaches including re-implantation of irradiation-devitalized tumor bone and autoclaving-devitalized tumor bone; G3 (Group 3), non-devitalized LSS group: LSS with reconstruction approaches including prosthetic replacement, allograft transplantation, and vascularized fibula autograft implantation. For each group, the average duration of DFS, the percentage of actuarial 5-year continuous disease free survival (CDFS), local recurrence, and the post-operative complications were calculated and analyzed. Statistical comparison was made with a-priori contrasts as follows (G1–G2), (G1–G3), (G2–G3) and (G1–G2 and G3 combined: Amputation vs. LSS).

Statistical analysis was performed using SPSS 10.0 software. The likelihood ratio chi-square test was used for significance testing. Survival rate was analyzed using the Kaplan-Meier method, and the differences in survival rates were compared using the log-rank test. The level of significance was set at P<0.05.

Our studies were approved by the ethics committee of Shanghai Tenth People's Hospital and the School of Medicine at Tongji University (Shanghai, China). Informed consent was not needed since the data were analyzed anonymously. The ethics committee specifically waived the need for consent.

## Results

Until December 2008, all 58 patients were followed up for ranging from 6 to 16 years (median 10.8 years). During the follow-up, the clinical information regarding the DSF, local recurrence, and the post-operative complication including infection, fracture, and non-union were recorded and used for analysis throughout this study.

**Figure 1:**
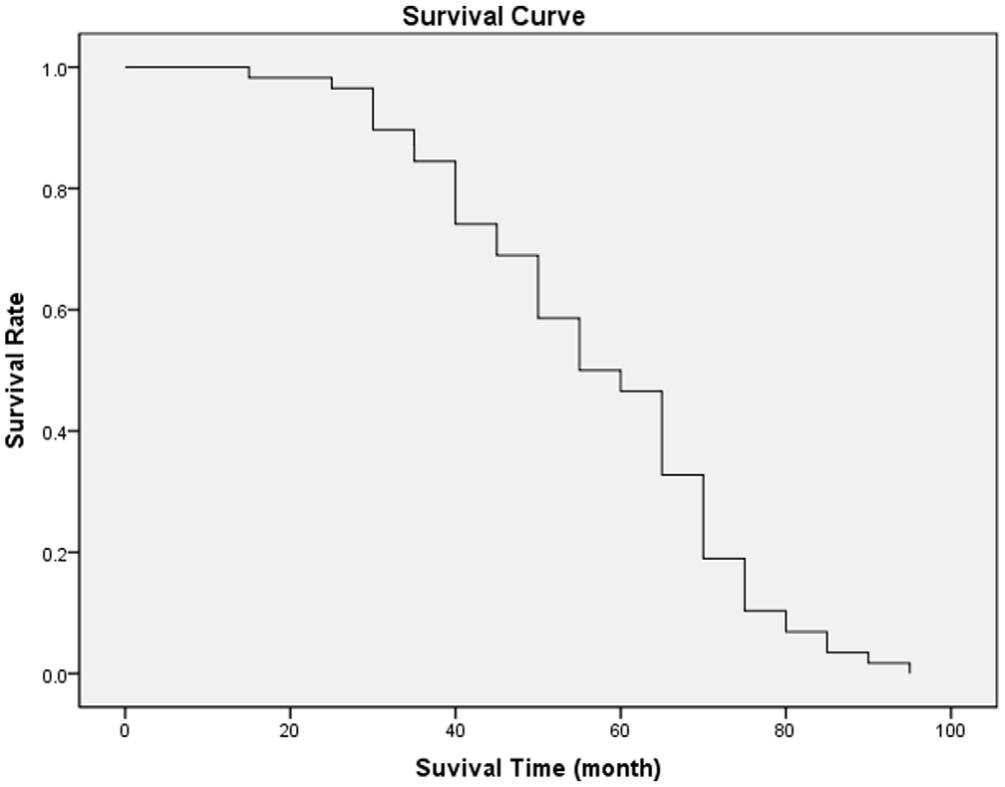
Kaplan-Meier survival curve of 58 patients with osteosarcoma.

The average duration of DFS was 53.3±17.8 months and the actuarial 5-year continuous disease free survival (5-year CDFS) was 46.5%. The survival curve monitored for up to 92 months following the surgery is shown in Figure 1. The overall percentage of local recurrence and post-operative complications including infection, fracture, and nonunion were 13.8% (8/58) and 32.8% (19/58), respectively.

**Figure 2:**
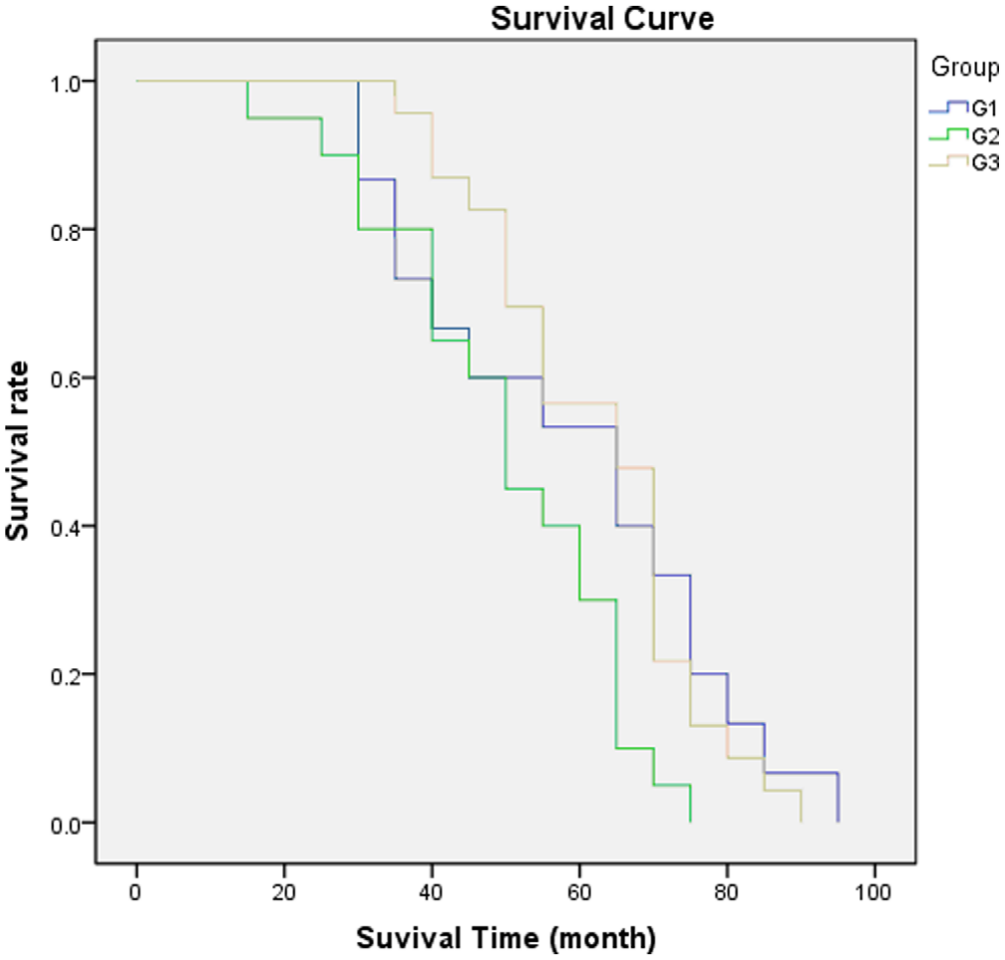
Comparison of Kaplan-Meier survival curves between amputation (G1), devitalized LSS (G2), and non-devitalized (G3) groups. Log-rank tests showed that disease-free survival was significantly different between G1 and G2 (P = 0.049) and G2 and G3 (P = 0.005) and no difference in G1 and G3 was found (P = 0.946).

The total 58 patients has been divided into three different groups (G1 to G3) based on the treatment protocol they received as described above. The survival curves of three groups (G1, G2, and G3) were generated individually and shown in Figure 2. The statistical analysis (log-rank test) was conducted thereafter and found that there was no pronounced difference between G1 and G3 (*P* = 0.946), and these two groups had significantly higher DFS than G2 (*P* = 0.049 between G1 and G2, and *P* = 0.005 between G2 and G3). For all 3 groups (G1, G2, and G3), the percentages of 5-year CDFS, local recurrence, and post-operative complications were calculated and compared statistically between different groups (Table 1). The three parameters for G1, G2, and G3 are respectively: the percentages of 5-year CDFS 53.3%, 30.0%, and 56.5%; the percentage of local recurrence 6.7%, 25.0%, and 8.7%; the percentage of complication 6.7%, 55%, and 30.4%. The comparison and statistical analysis showed that the difference in the percentage of complication after surgery was found between G1 and G2 (P = 0.003), G2 and G3 (P = 0.079), and G1 and G3 (P = 0.103), but only significant for G1 and G2 (P<0.05). The percentages of 5-year CDFS and local recurrence were also more or less different between the three groups, but not statistically significantly with P>0.05 (shown in Table 1).

**Table 1:** Comparison of outcomes among amputation (G1), devitalized LSS (G2), and non-devitalized LSS groups (G3).

Percentage(%)	5-year CDFS			Local Recurrance			Post-operative Complication		
	G1 (n = 15)	G2 (n = 20)	G3 (n = 23)	G1 (n = 15)	G2 (n = 20)	G3 (n = 23)	G1 (n = 15)	G2 (n = 20)	G3 (n = 23)
	53.3 (8)	30 (6)	56.5 (13)	6.7 (1)	25 (5)	8.7 (2)	6.7 (1)	55 (11)	30.4 (7)

Chi-squaretest	P value								
G1	NA	0.163	0.847	NA	0.154	0.073	NA	0.003	0.079
G2	0.163	NA	0.081	0.154	NA	0.821	0.003	NA	0.103
G3	0.847	0.081	NA	0.073	0.821	NA	0.079	0.103	NA

**Figure 3:**
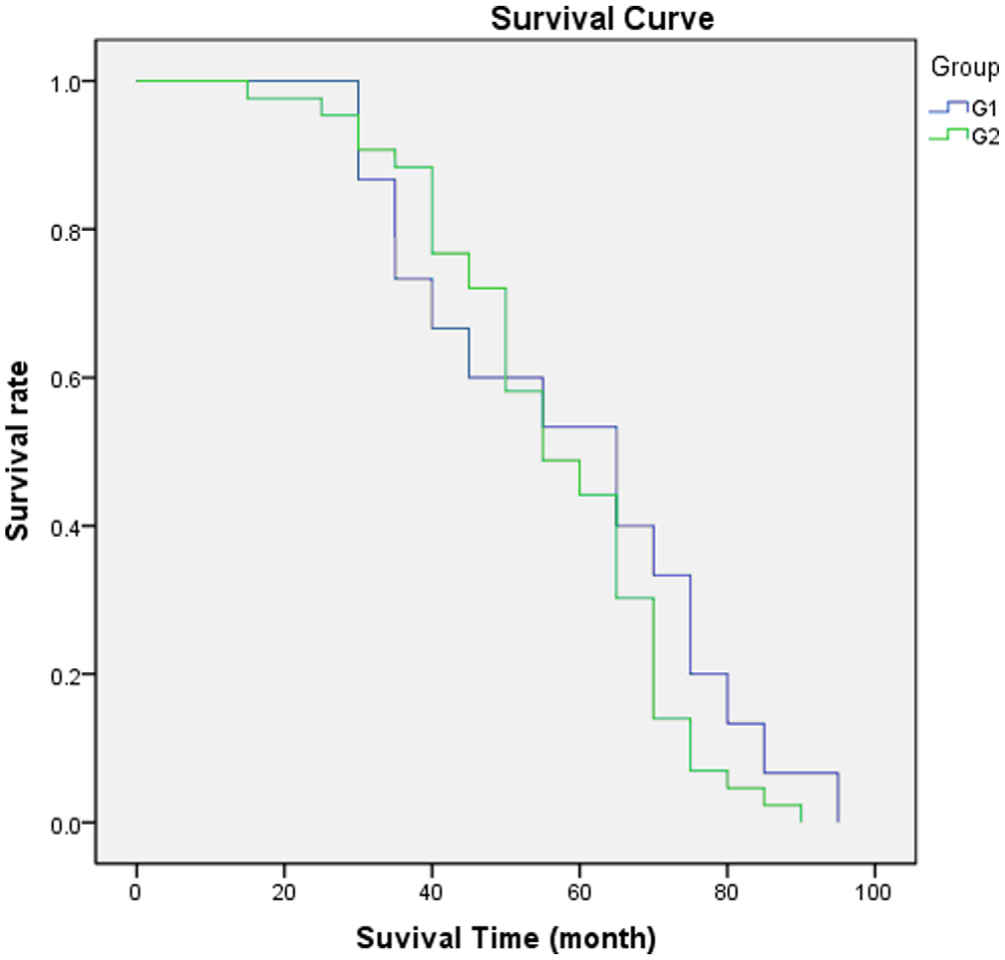
Comparison of Kaplan-Meier survival curves between amputation group and LSS treated groups (G2 and G3 combined). The log-rank test suggested that DFS was not significantly different between these two groups (*P* = 0.313).

When G2 and G3 were combined as one group, the treatment outcomes in terms of the percentages of 5-year CDFS, local recurrence, and post-operative complications were derived and compared to G1 (Table 2), which represents the comparison between two major surgery protocols: amputation (G1) and LSS (G2 and G3 combined). Log-rank tests showed no significant difference in DFS was observed from the survival curves (Figure 3, P = 0.313). The percentages of CDFS, local recurrence, and post-operative complications for G2 and G3 combined were 44.2%, 16.3%, and 41.8%, respectively, which were different from the values for G1, but only significantly in terms of post-operative complication (P = 0.012).

**Table 2:** The outcome analysis of LSS treated group (G2 and G3 combined, n = 43).

5-year CDFS		Local Recurrence		Post-operative Complication	
Percentage(%)	P value*	Percentage(%)	P value	Percentage(%)	P value
44.2 (9)	0.541	16.3 (7)	0.353	41.8 (18)	0.012

Note: P value was analyzed by comparison to G1.

When the LSS group (G2 and G3) were divided further into the 5 subgroups, irradiation LSS, autoclaving LSS, prosthetic LSS, allograft LSS, and autograft LSS, based on the reconstruction procedure following LSS, the percentages of 5-year CDFS, local recurrence, and post-operative complication were remarkably different. The percentages of 5-year CDFS were 25.0%, 37.5%, 54.5%, 62.5% and 50% in the irradiation, autoclaving, prosthetic, allograft, and autograft subgroup, respectively. The percentages were 33.3%, 12.5%, 9.0%, 12.5% and 0% for local recurrence, and 41.7%, 75.0%, 18.1%, 62.5% and 0% for post-operative complication. No statistical analysis was made due to the insufficient sample size in some subgroups.

As noted above, the percentage of post-operative complication was significantly higher in the LSS group (41.8%) compared to the amputation group (6.7%, *P* = 0.012). The main complication after LSS was infection, fracture (featuring seroma/serous leak), and non-union of bone. The overview of the complication occurred after surgery was summarized here: Eight patients (two in each of irradiation and prosthetic subgroups, three in autoclaving subgroup, one in allograft subgroup) experienced infections post-operatively, which occurred in the proximal tibia (n = 5) or the distal femur (n = 3) 7–60 days post-surgery; Fractures of grafted bones occurred in the distal femur and the proximal tibia of six patients within the irradiated and autoclaved subgroups (n = 4) and the allograft subgroup (n = 2), 1–3 years after surgery; Non-union of bone occurred in the proximal tibia of 4 patients, among whom two were in the irradiated and autoclaved subgroups and two were in the allograft subgroup.

## Discussion

The treatment of osteosarcoma has been advanced dramatically over the last two decades by applying LSS combined with adjuvant chemotherapy.Various surgeries applied along with the chemotherapeutic drugs including methotrexate, adriamycin, and cisplatin has greatly increased the survival rates of patients and the life quality of patients has also been further improved by the replacement of amputation with LSS [7]. Radical resection, or at least wide resection, is recommended for stage II osteosarcoma [8]–[10]. In our practice, wide resection plus reconstruction along with chemotherapy was routinely performed for the treatment of osteosarcoma. Amputation is only considered in patients whose vessels and nerves have been widely affected by the tumor(s) [11].

Given the differences in prevalence, recurrence rates and post-operative complications at different sites, our study only included the cases with tumor at the particular knee joint area. A period of five or more years of disease-free survival is defined as “cured” with respect to osteosarcoma [12], [13]. In our study, Kaplan-Meier analysis demonstrated that the overall survival curve among all 58 patients reached about 50% after three to five years postoperatively. Amputation and LSS surgery could result in the similar disease-free survival rate. However, when compared the disease-free survival rate of the patients treated with amputation surgery to the LSS treatments plus different reconstruction procedures (devitalized: irradiation and autoclaving, and non-devitalized: prosthetic, allograft, and autograft), both of amputation and non-devitalized LSS group had similar survival rates, both of which were better than non-devitalized LSS group. These data suggest that the procedure for reconstruction following LSS surgery is critical for the outcome of the treatment applied for osteosarcoma.

The previous report shows that the relapse and metastasis of osteosarcoma occurs typically 1–2 years following surgery [14]. The recurrence rate after LSS for osteosarcoma is usually 10–20% [15], [16]. As expected, the recurrence rate in all LSS treated patients was 16.3% in this study, which is higher than that in amputation treated group (G1, 6.7%). When looking into the result further, the reconstruction procedure was found to affect the local recurrence substantially following LSS surgery: in G2 (devitalized LSS group), the recurrence was 25% and 8.7% for G3 (non-devitalized LSS group). More specifically, the percentage of local recurrence after surgery varied much by the different reconstruction procedure ranging from 33.3% for irradiation to as low as 0% in autograft subgroup (the case number is only 4). The percentage of 5-year CDFS was also lower in the irradiated and autoclaved subgroups (<50%) and relatively high in the prosthetic (54.5%) and allograft (62.5%) subgroups, which leads to the lower percentage of 5-year CDFS in devitalized LSS group (30.0%) in relative to non-devitalized LSS group (56.5%) and amputation group (53.3%), even not significantly. Once again, the reconstruction approach was approved to be essential for the long-term outcome including both 5-year CDFS and local recurrence of the LSS for the treatment of osteosarcoma. The underlined reason for lower 5-year CDFS rate and higher recurrence rate in irradiation LSS subgroup could be explained by the previous research work: osteosarcomas was reported not sensitive to radiotherapy and the previous research [17], [18] has shown that *in vitro* osteosarcoma cells could only be devitalized by a radiation dose of 60,000–100,000 Gy. The medical equipment available at present (radiation dose: 30–50 Gy) is not able to produce such high doses of rediation. Therefore, irradiation is not recommended for devitalization. Instead, boiling for 30 min or soaking in 95% (v/v) ethanol for 30 min is preferred. Other *in situ* devitalization of tumor-containing bones using microwave heliotherapy [19] or high intensity focused ultrasound [20] has also been well developed and reported.

Although LSS has become one major option for the treatment of osteosarcoma, post-operative complications including infections, fractures, non-union of bones, and loosening of prostheses are still of intensive concern. The incidence of LSS-related complications was from 31.4–63.0% [21]–[23]. In our study, the incidence of post-operative complications was 41.8% in the LSS group, which was much higher than that in the amputation group (6.7%), and devitalized reconstruction procedure (55.0%) had more chance of post-operatively complication than non-devitalized procedure (30.4%). Among post-operative complications, infection was the most common complication, accounting for 61.5% of the cases with complication. Post-operative infections are usually quite difficult to treat and amputation is often required eventually. In our study, infections were more common in the irradiated, autoclaved and allograft subgroups.This suggests that the problems including graft rejection, virus infection, and donor-recipient mismatch need to be better addressed and the prevention and treatment of post-LSS infections remains a challenge. To more effectively prevent infections, chemotherapy and radiotherapy are usually performed two weeks before surgery, followed by routine administration of prophylactic antibiotics one day before and during surgery (Note that the patients included in this study were not treated by radiotherapy). Post-operative fracture, usually occurred 1–2 years after surgery, ranked second among the complications in our series, especially in the devitalized LSS group(20% of incidence). The incidence of fracture was especially high in the proximal tibia due to the severe impairment of bone substances after devitalization, poor blood supply in the proximal tibia, and heavy load on the implants [24]. Bone cement filling and fixation with interlocking intramedullary nails during the re-implantation of devitalized bones may achieve lesser stress-shielding effects and lower post-operative fracture rates. Non-union of bones is often seen in the proximal tibia, especially in patients who have undergone implantation of allografts or autogenous bone grafts, and may also be relevant when poor blood supply is occurring. Similarly, once a non-union occurs, prosthesis replacement is usually required. Different techniques have been proposed to reduce complications and improve functions of the affected extremities [25]–[27].

Collectively, LSS combined with adjuvant chemotherapy has the comparable survival rates of patients with osteosarcoma at knee joint area, especially by LSS with non-devitalized reconstruction procedure to those of amputation. Even the incidence of the post-operative complication is higher, this treatment protocol can provide the advantage for improving the life quality of patients over amputation protocol and the complications can be minimized by using chemotherapy with antibiotics before and after surgery, choosing the most appropriate reconstruction procedure following LSS (recommend prosthetic for proximal tibia), and better prevention and post-operative care to the patients. Our study shows that the procedure for reconstruction following LSS surgery is critical for the outcome of the treatment applied for osteosarcoma. At the current stage, the non-devitalized procedure including irradiation is not recommended for bone reconstruction in LSS considering the lower percentage of 5-year CDFS and higher incidence of local recurrence and post-operative complication associated with this procedure. Autoclaving can be employed if the devitalized procedure has to be considered. Even amputation has good DFS, lower local recurrence and post-operative complication, it will be only chosen in patients whose vessels and nerves have been widely affected by the tumor considering the long-term life quality of patient.
